# Circulating CARMN for acute myocardial infarction prognosis and its incremental effect when combined with conventional biomarkers

**DOI:** 10.3389/fcvm.2026.1881009

**Published:** 2026-10-21

**Authors:** Zhirui Chen, Jue Chen, Jieming Zhou, Zhenxing Yao

**Affiliations:** Emergency Medicine Center, Affiliated Hospital of Guangdong Medical University, Zhanjiang, Guangdong, China

**Keywords:** AMI, CARMN, cTnI, echocardiography, MACE

## Abstract

**Background:**

This study aims to explore the clinical value of lncRNA CARMN in acute myocardial infarction (AMI) patients.

**Methods:**

The study subjects included 156 AMI patients and 59 healthy controls. The serum CARMN levels were detected by real-time quantitative polymerase chain reaction (RT-qPCR). The correlation between CARMN and myocardial injury markers, as well as cardiac ultrasound indicators by Pearson analysis. Kaplan–Meier and Cox regression were employed to screen out the independent influencing factors for MACE in AMI patients, and a nomogram was constructed.

**Results:**

In patients with AMI, the serum CARMN level is decreased. CARMN level correlated with cTnI, CK-MB, Myo, LVEF. A total of 156 patients with AMI were enrolled and followed up for 6 months, among whom 60 patients experienced MACE. Lower CARMN levels were associated with a higher incidence of MACE. The AUC of the combined model for predicting MACE reaches 0.867, and the nomogram has good calibration and high clinical net benefit.

**Conclusion:**

Serum CARMN combined with myocardial injury markers and ultrasound indicators, may help predict MACE risk in patients with AMI.

## Introduction

Acute myocardial infarction (AMI) is mainly caused by interruption of blood flow in the coronary arteries, resulting in ischemic and necrotic damage to the heart muscle (1). AMI is a serious cardiovascular disease, with typical symptoms being severe and oppressive chest pain (2). In clinical practice, AMI is mainly diagnosed through the combination of clinical symptoms, electrocardiogram and myocardial markers (3, 4). However, even if patients receive timely treatment, there are still significant differences in their prognosis (5). Early identification of the risk factors for poor prognosis in AMI is of vital importance for optimizing the treatment of AMI.

Myocardial markers commonly used in AMI diagnosis include cardiac troponin I (cTnI), creatine kinase-MB (CK-MB), and myoglobin (Myo) (6, 7). Among them, cTnI is a specific regulatory protein for cardiac muscle cells (8). When irreversible necrosis occurs in cardiac muscle cells, cTnI is rapidly released into the bloodstream (9, 10). Currently, it is recognized as the gold standard marker for evaluating AMI (11). CK-MB is one of the isoenzymes of creatine kinase and reaches its peak approximately 24 h after myocardial infarction (12). Although Myo has lower myocardial specificity relative to cTnI, it still plays an important role in the diagnosis and prognosis assessment of AMI (13). Myo is a small molecule oxygen-binding protein present in both cardiac and skeletal muscles (14). It has the characteristics of a small molecular weight and rapid release. Myo is usually used in combination with other markers to improve the accuracy of diagnosis (15).

Long non-coding RNAs (LncRNAs) represent an important class of non-coding RNAs that exhibit stable persistence in the blood and are easily detectable (16). Therefore, lncRNAs are considered among the most promising biomarkers currently available (17). Zuo et al. confirmed that the combination of lncRNA LINC00472 with serum CK-MB/cTnI levels may enhance the diagnostic value of CK-MB or cTnI in the early diagnosis of AMI (18). Tayae et al. suggests that HIF1A-AS2 may be a potential early diagnostic biomarker of AMI (19). LncRNA CARMN is located on human chromosome 19 and is mainly expressed in cardiac tissues (20). In both mouse and human atherosclerotic plaques, CARMN colocalized with vascular smooth muscle cells (VSMCs) and was expressed in the nucleus (21). Studies have shown that knocking down CARMN could promote the dedifferentiation of VSMCs via downregulation of miR-145 and miR-143, thereby accelerating atherosclerosis progression (22). Another study discovered that the high-enriched CARMN in VSMCs significantly decreased with the occurrence of chronic limb ischemia (23). Our study aims to explore the correlation between the expression level of serum CARMN and myocardial injury markers, as well as electrocardiogram parameters. It also aims to construct a predictive model that can forecast the prognosis progression of AMI patients to major adverse cardiovascular event (MACE), providing new ideas and experimental basis for clinical assessment of the poor prognosis of AMI patients.

## Materials and methods

### Sample collection

This retrospective cohort study consecutively enrolled 156 patients diagnosed with AMI who underwent percutaneous coronary intervention (PCI) at Guangdong Medical University Affiliated Hospital from January 2023 and January 2025. The details of the patient’s treatment can be found in Supplementary Table S1. Additionally, 59 healthy individuals who underwent physical examinations during the same period were included. Inclusion criteria: (1) In accordance with the expert consensus diagnostic criteria for AMI; (2) The time from onset to admission was ≤ 72 h; (3) Age was over 18 years; (4) Complete clinical data. Exclusion criteria: (1) Complicated with other severe heart diseases, infectious diseases, malignant tumors, severe liver and kidney dysfunction, etc.; (2) Patients with mental disorders, cognitive dysfunction, and unable to cooperate in data collection. This study complies with the principles in the Helsinki Declaration and has been approved by the Ethics Committee of Affiliated Hospital of Guangdong Medical University. All participants also gave informed consent. Collection of venous blood samples and completion of echocardiography at 24 h after PCI (24).

### Data collection

Collect the baseline data of the subjects, including age, gender, body mass index (BMI), and smoking history (daily smoking of ≥1 cigarette and lasting for more than 1 year). Also record whether the subjects have a history of diabetes and hypertension. Collect the laboratory indicators of the subjects, such as total cholesterol (TC), triglycerides (TG), high-density lipoprotein cholesterol (HDL-C), and low-density lipoprotein cholesterol (LDL-C).

### Myocardial injury markers

Collect 3–5 mL of fasting venous blood from the subjects, and centrifuge to separate the upper serum layer. The levels of cTnI, CK-MB and Myo in the patient’s blood were measured using the fully automated chemiluminescence immunoassay analyzer Roche Cobas e601 (Roche, Basel, Switzerland).

### Echocardiography

The echocardiogram was examined using the Vivid E95 color Doppler ultrasound instrument (General Electric Company, USA). During the examination, the patient was placed in a supine position and a comprehensive scan was conducted on the chest and heart area. The left ventricular volume and ejection fraction were measured using the Simpson two-plane method. The instrument automatically calculated the left ventricular ejection fraction (LVEF). Each indicator was measured for three consecutive cardiac cycles and the average value was taken to accurately assess the overall systolic function of the left ventricle.

### RT-qPCR analysis

Total RNA was extracted from 200 μL serum samples using the TRIzol method. The concentration and purity of the RNA were determined using a NanoDrop 2000 spectrophotometer (Invitrogen, USA). The sample was considered qualified when the A260/A280 ratio ranged from 1.8 and 2.0. The A260/A230 ratio was also recorded to evaluate residual salt and organic reagent contamination. According to the instructions of the reverse transcription kit (TIANGEN, China), 1 μg of RNA was reverse transcribed into cDNA. RT-qPCR was performed using the SYBR Premix Ex TaqⅡ kit (Thermo Fisher, USA) on an ABI 7500 real-time fluorescence quantitative PCR instrument (Applied Biosystems, USA). *β*-actin was used as the internal reference gene, based on conventional literature practice. To preliminarily verify its applicability, we compared *β*-actin Ct values between the patient and control groups, and no significant difference was observed (*P* > 0.05). CARMN expression was calculated using the 2^−*ΔΔ*Ct^ method. Three technical replicates were set for each sample, and the average Ct value was used for analysis. *Δ*Ct was defined as Ct *_CARMN_*-Ct *_β−actin_*. The mean *Δ*Ct of healthy controls served as the calibrator for *ΔΔ*Ct calculation. The inter-assay coefficient of variation (CV) of Ct values for target and reference genes was controlled below 5%. CARMN, Forward GTGCTGCATCTCTGGTCAGT, Reverse AGACCGCTTGGGTCTCAATG; *β*-actin, Forward GGTAAAAGGCAGAGTGGCCT, Reverse GGGGTGTCACTGCTTCTCAA.

### Follow-up

The patients were followed up for a period of 6 months. The follow-up began immediately after AMI patients underwent PCI surgery and continues until the occurrence of a MACE or the end of the pre-specified 6-month follow-up period. The definition of major adverse cardiovascular events (MACE) is a composite endpoint consisting of all-cause death, non-fatal myocardial infarction, or non-fatal stroke, heart failure or malignant arrhythmia, with the occurrence of any one of these events being taken as the criterion (25, 26). During follow-up, all-cause death occurred in 5 patients, non-fatal myocardial infarction in 9, non-fatal stroke in 13, heart failure in 18, and malignant arrhythmia in 15. AMI patients were categorized into MACE and non-MACE groups according to the occurrence of MACE.

### Statistical methods

Statistical analyses were carried out using SPSS 26.0 (IBM Corp., Armonk, NY, USA) and GraphPad Prism 9.0 (GraphPad Software, San Diego, CA, USA). Normally distributed continuous variables were compared between two groups using the independent sample t-test and among multiple groups using one-way analysis of variance (ANOVA). Non-normally distributed continuous variables were compared between two groups using the Mann–Whitney U test. Categorical variables were conducted between groups using the chi-square test. The correlation between CARMN levels and myocardial injury markers, as well as cardiac ultrasound indicators, was analyzed using Penson. Receiver Operating Characteristic (ROC) curve evaluated the value of CARMN in the diagnosis and prognosis assessment of AMI. Kaplan–Meier survival curves were constructed to show MACE incidence among AMI patients stratified according to CARMN expression. Cox regression analysis identified potential risk factors for the occurrence of MACE in AMI patients. Multicollinearity between variables was assessed by variance inflation factor (VIF) and pairwise correlation coefficients. The VIF values of the remaining predictors (cTnI, CK-MB, CARMN, LVEF) all ranged from 1.072 to 1.161, indicating no significant multicollinearity. A nomogram, calibration curve, and decision curve were drawn using R language. A *P* value < 0.05 was considered statistically significant.

## Results

### Baseline characteristics

As shown in Table 1, there were no statistically significant differences between patients with AMI and the control group in terms of gender, age, BMI, smoking history, history of hypertension, history of diabetes, and lipid indicators (TC, TG, HDL-C, LDL-C) (all *P* > 0.05). In terms of myocardial injury markers, the values of cTnI, CK-MB, and Myo in the AMI group were all higher than those in the control group (all *P* *<* 0.001). In terms of cardiac ultrasound indicators, the LVEF in the AMI group was significantly lower than that in the control group (*P* *<* 0.001).

**Table 1:** Baseline data and laboratory indicators of the participants.

Variables		Control (*n* = 59)	AMI (*n* = 156)	*P* value
Gender	Male	25	77	0.360
	Female	34	79	
Age (year)		51.00 (9.00)	52.00 (11.00)	0.119
BMI (kg/m^2^)		23.72 (4.57)	23.05 (4.84)	0.214
Smoking history		15	37	0.794
Hypertension		11	36	0.483
Diabetes		8	19	0.785
TC (mmol/L)		4.08 (1.28)	3.87 (1.10)	0.316
TG (mmol/L)		0.87 (0.37)	0.90 (0.34)	0.445
HDL-C (mmol/L)		1.66 (0.55)	1.61 (0.40)	0.553
LDL-C (mmol/L)		2.34 (0.89)	2.51 (0.95)	0.379
Myo (ng/mL)		58.53 (6.30)	87.17 (10.41)	<0.001
cTnI (µg/L)		0.45 (0.18)	1.72 (0.41)	<0.001
LVEF (%)		63.23 (6.73)	51.88 (9.58)	<0.001
CK-MB (ng/mL)		2.92 (0.87)	12.22 (1.29)	<0.001

BMI, body mass index; TC, total cholesterol; TG, triglycerides; HDL-C, high-density lipoprotein cholesterol; LDL-C, low-density lipoprotein cholesterol; Myo, myoglobin; cTnI, cardiac troponin I; CARMN, cardiac mesoderm enhancer-associated non-coding RNA; LVEF, left ventricular ejection fraction; CK-MB, creatine kinase-myocardial band.

### Serum CARMN levels and its diagnostic value

CARMN was significantly reduced in AMI patients compared to controls (Figure 1A, *P* *<* 0.05). The ROC curve showed that serum CARMN could distinguish AMI patients from healthy controls with an area under the curve (AUC) of 0.849, and when the cutoff value was 0.865, the sensitivity was 74.36% and the specificity was 83.05% (Figure 1B, *P* *<* 0.05).

**Figure 1:**
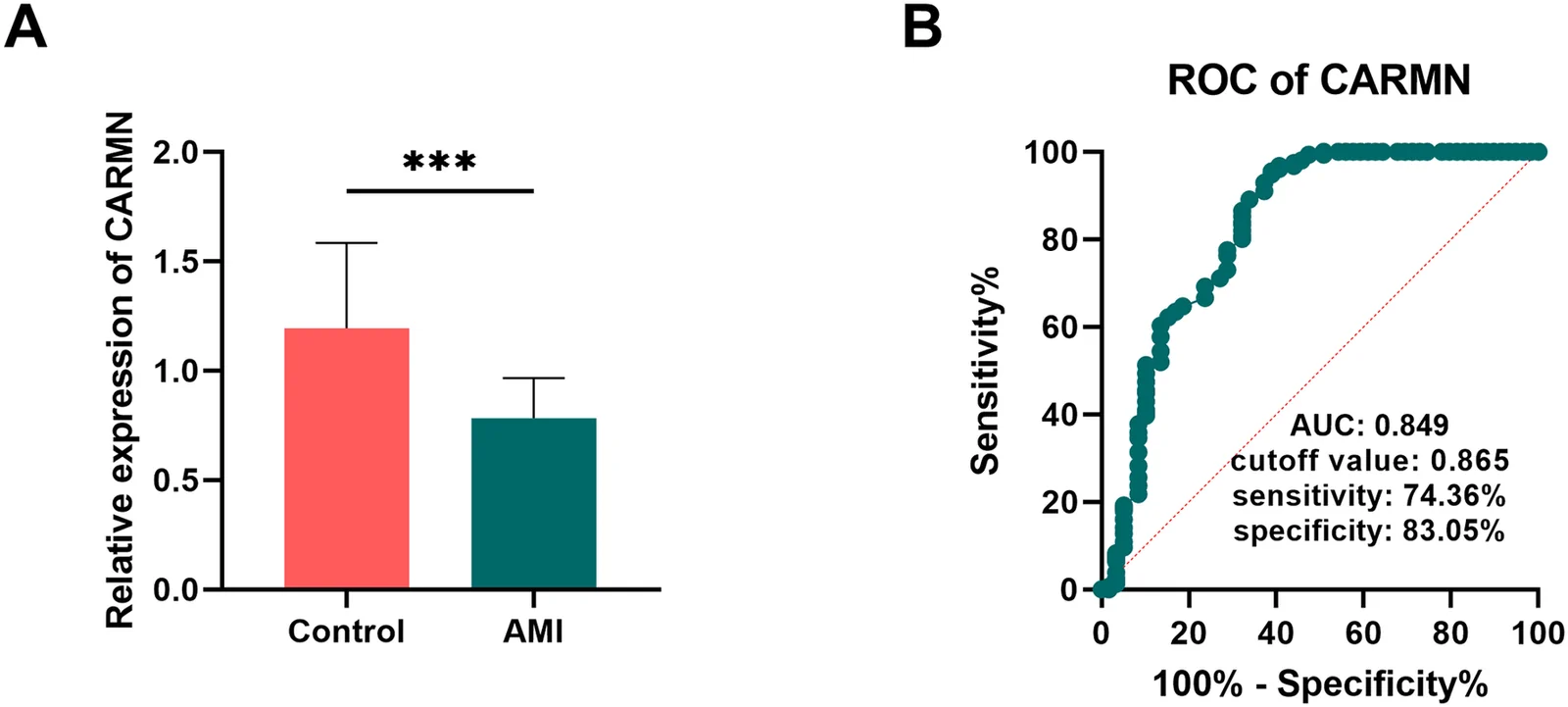
Expression level of CARMN in the serum of AMI patients. **(A)** Comparison of the expression level of CARMN between the control group and the AMI patients’ serum. **(B)** Receiver operating characteristic (ROC) curve of CARMN for differentiating AMI patients from the control group.

### Serum CARMN levels are correlated with myocardial injury markers and cardiac function

Pearson correlation analysis was performed to assess the correlations between CARMN expression and myocardial injury markers (cTnI, CK-MB, Myo) as well as cardiac systolic function in patients with AMI. As shown in Figure 2, serum *CARMN* expression exhibited significant negative correlations with cTnI (Figure 2A, r = −0.623), CK-MB (Figure 2B, r = −0.729), Myo (Figure 2C, r = −0.747). In addition, a significant positive correlation was observed between serum *CARMN* levels and LVEF (Figure 2D, r = 0.672).

**Figure 2:**
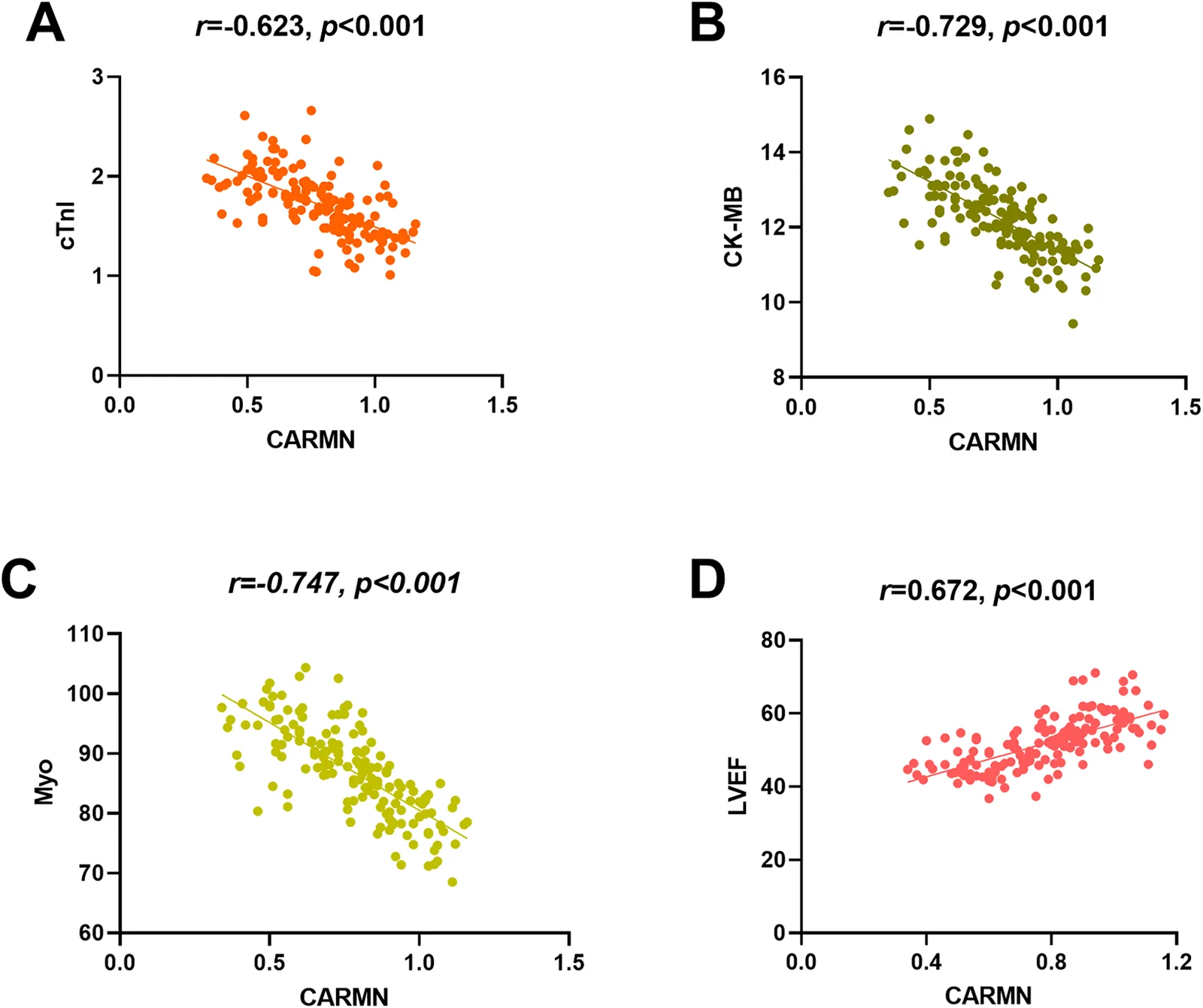
Correlation analysis of serum CARMN expression levels and myocardial injury markers. **(A)** The expression level of CARMN was significantly negatively correlated with cTnI (*r* = −0.623, *P* < 0.001). **(B)** The expression level of CARMN was significantly negatively correlated with CK-MB (*r* = −0.729, *P* < 0.001). **(C)** The expression level of CARMN was significantly positively correlated with Myo (*r* = −0.762, *P* < 0.001). **(D)** The expression level of CARMN was significantly positively correlated with LVEF (*r* = 0.672, *P* < 0.001).

### Serum CARMN can predict the occurrence of MACE

Based on the median CARMN expression in AMI patients, the patients were divided into high-expression group (*n* = 78) and low-expression group (*n* = 78). The CARMN low-expression group exhibited a higher incidence of MACE, which was indicative of poor prognosis among AMI patients (Figure 3A, Log-rank *P* *<* 0.001). Furthermore, we compared the expression of CARMN in patients with MACE. The results revealed that CARMN expression levels were significantly lower in patients who developed MACE (*n* = 60) compared to those who did not (*n* = 96) (Figure 3B, *P* *<* 0.05).

**Figure 3:**
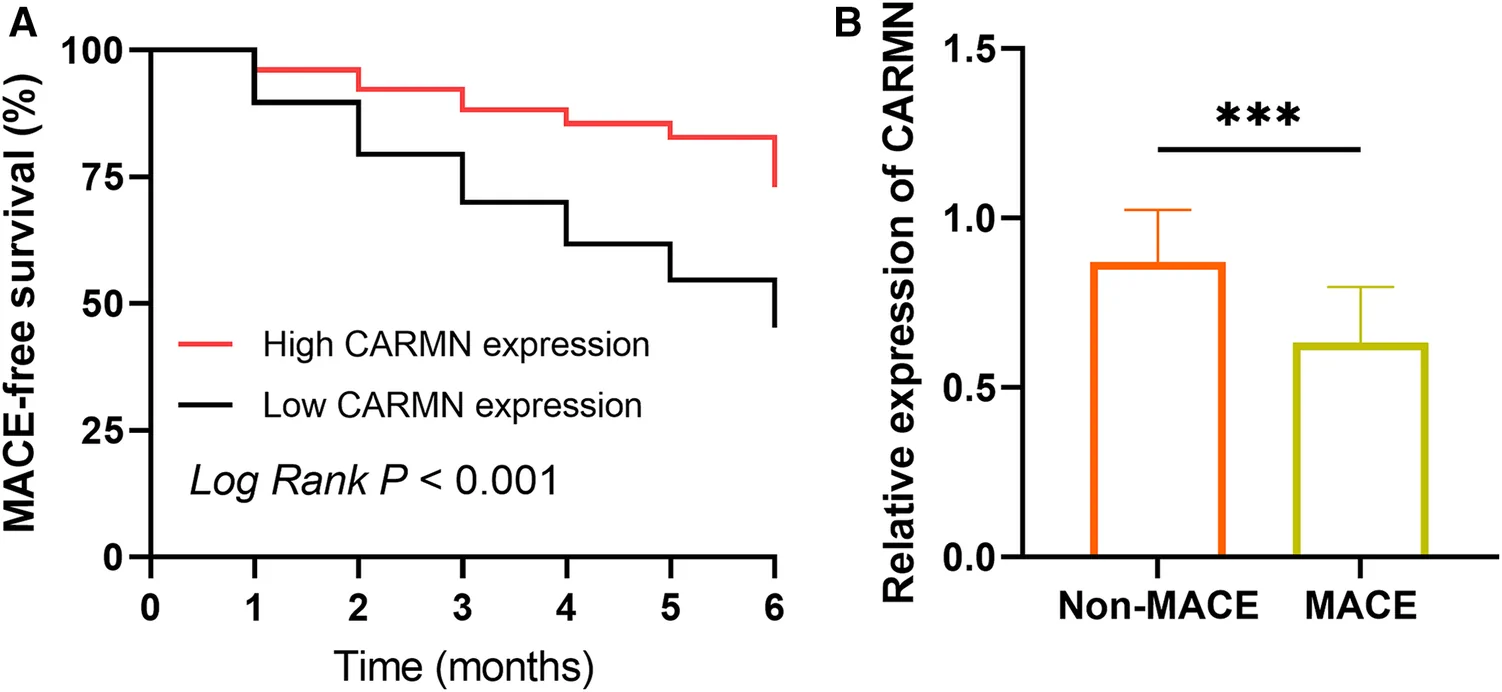
The relationship between CARMN and the occurrence of MACE within 6 months. **(A)** The correlation between CARMN levels and the survival period of patients without MACE. **(B)** The expression of CARMN in patients with MACE.

### Cox regression analysis of the influencing factors of MACE

In addition, we also conducted univariate and multivariate Cox regression analyses to determine the independent risk factors for the occurrence of MACE. During the 6-month follow-up, 60 patients experienced MACE and 11 patients were censored. Results from the univariate Cox regression analysis indicated that cTnI, CARMN, LVEF, and CK-MB were potential influencing factors for the occurrence of MACE in AMI patients. The collinearity analysis indicated that there was no significant multicollinearity among cTnI, CK-MB, CARMN and LVEF. Two nested multivariable Cox models were then constructed. Model-A incorporated conventional clinical covariates (age, diabetes mellitus, cTnI, CK-MB, and LVEF). CARMN was further added into Model-B based on Model-A. In the fully adjusted Model-B, cTnI (HR = 4.878, 95% CI: 2.026–11.747, *P* < 0.001), CK-MB (HR = 1.823, 95% CI: 1.334–2.491, *P* < 0.001), low expression of CARMN (HR = 0.049, 95% CI: 0.012–0.200, *P* < 0.001) and decreased LVEF (HR = 0.910, 95% CI: 0.868–0.954, *P* < 0.001) were independent risk factors for MACE in AMI patients (Table 2).

**Table 2:** Cox regression analysis.

Variable	Univariate analysis			Multivariate analysis-A (without CARMN)			Multivariate analysis-B (with CARMN)		
	HR	95% CI	*P*	HR	95% CI	*P*	HR	95% CI	*P*
Gender	1.323	0.795–2.200	0.281	-	-	-			
BMI	1.007	0.922–1.099	0.880	-	-	-			
Smoking	0.804	0.427–1.513	0.499	-	-	-			
Hypertension	1.223	0.682–2.195	0.500	-	-	-			
TC	1.208	0.837–1.745	0.313	-	-	-			
TG	0.613	0.213–1.766	0.365	-	-	-			
HDL-C	0.897	0.359–2.240	0.815	-	-	-			
LDL-C	0.986	0.630–1.545	0.952	-	-	-			
Myo	1.009	0.975–1.045	0.605	-	-	-			
Age	0.992	0.960–1.024	0.614	0.997	0.965–1.029	0.838	1.008	0.976–1.041	0.616
Diabetes	1.159	0.551–2.440	0.697	1.718	0.781–3.781	0.178	1.519	0.675–3.418	0.312
cTnI	6.809	2.760–16.796	<0.001	6.217	2.548–15.619	<0.001	4.878	2.026–11.747	<0.001
CARMN	0.011	0.003–0.039	<0.001	-	-	-	0.049	0.012–0.200	<0.001
LVEF	0.879	0.841–0.919	<0.001	0.900	0.859–0.942	<0.001	0.910	0.868–0.954	<0.001
CK-MB	2.491	1.893–3.278	<0.001	2.198	1.597–3.023	<0.001	1.823	1.334–2.491	<0.001

BMI, body mass index; TC, total cholesterol; TG, triglycerides; HDL-C, high-density lipoprotein cholesterol; LDL-C, low-density lipoprotein cholesterol; Myo, myoglobin; cTnI, cardiac troponin I; CARMN, cardiac mesoderm enhancer-associated non-coding RNA; LVEF, left ventricular ejection fraction; CK-MB, creatine kinase-myocardial band.

### ROC analysis of serum CARMN for predicting MACE

To evaluate the predictive value of each indicator for the occurrence of MACE in AMI patients, we conducted a ROC analysis (Figure 4). The results showed that the AUC of serum CARMN for predicting MACE was 0.847, with a cutoff value of 0.785, a sensitivity of 82.46%, and a specificity of 72.73%. The AUC of cTnI was 0.690, with a cutoff value of 1.715, a sensitivity of 84.12%, and a specificity of 70.71%. The AUC of CK-MB was 0.806, with a cutoff value of 12.33, a sensitivity of 75.44%, and a specificity of 74.75%. The AUC of LVEF was 0.794, with a cutoff value of 50.68%, a sensitivity of 70.18%, and a specificity of 72.73%. We further performed ROC curve analysis for the two prediction models.

**Figure 4:**
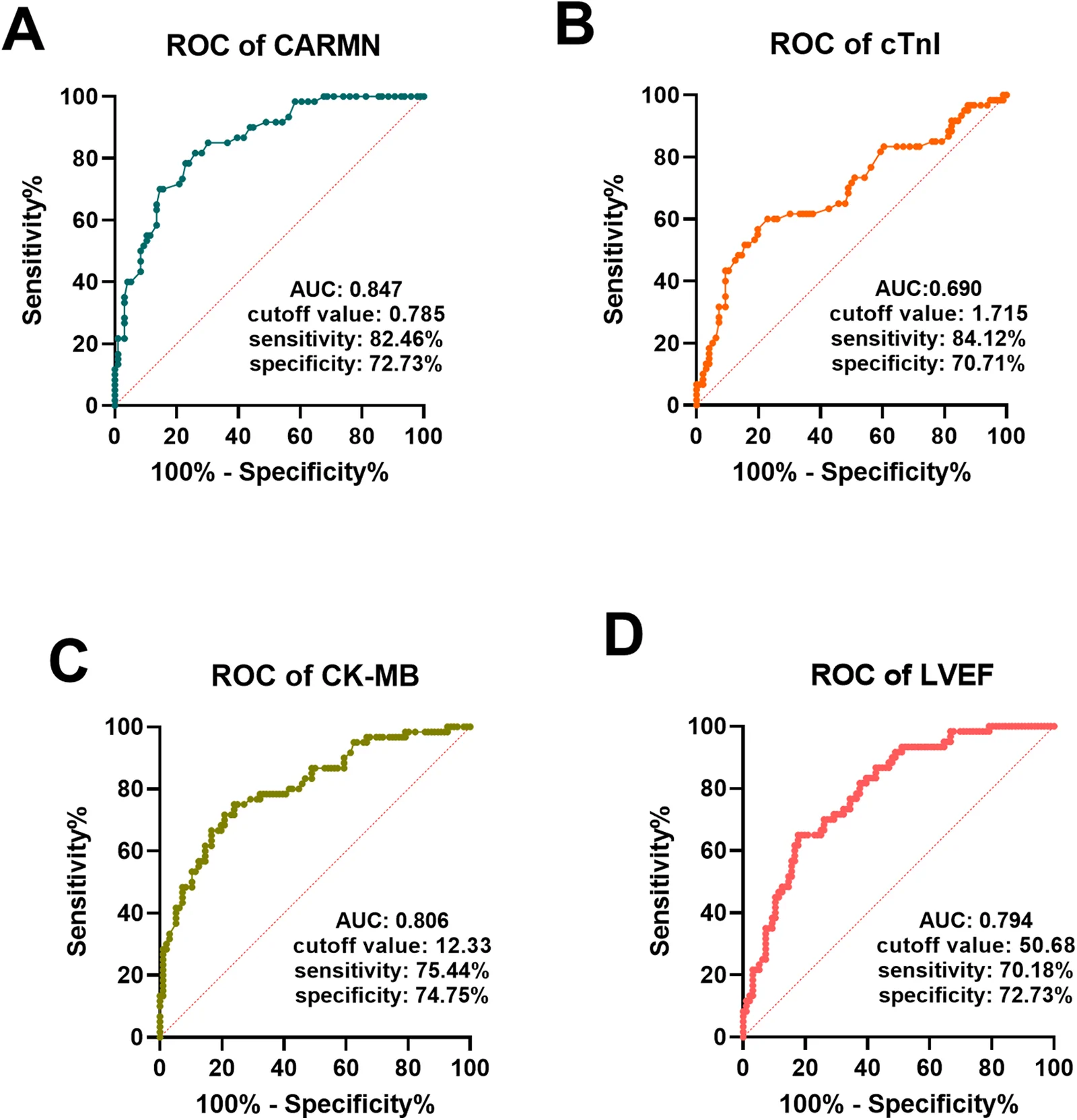
Comparison of the predictive efficacy of various indicators for the occurrence of MACE in AMI patients. **(A)** CARMN. **(B)** cTnI. **(C)** CK-MB. **(D)** LVEF.

### A CARMN-based nomogram for MACE risk prediction

Given the prognostic value of CARMN and the factors selected through Cox regression analysis, we constructed a nomogram model that comprehensively incorporated various variables to predict the risk of MACE in AMI patients (Figure 5A). Based on this line graph, we can predict the risk of MACE occurring within 6 months for AMI patients by using information such as CARMN, cTnI, LVEF and CK-MB levels of the patients. The ROC curve analysis showed that the AUC of the model was 0.867 (95% CI: 0.804–0.931) (Figure 5B). After internal validation using 1000 bootstrap resamples, the optimism-corrected C-index was 0.851. The calibration curve showed that the predicted probability of MACE occurrence by the nomogram was in good consistency with the actual observed probability, suggesting a good calibration degree of the model (Figure 5C). The decision curve analysis (DCA) revealed that within a wide range of risk thresholds, the prediction model based on this nomogram had a higher clinical net benefit compared to a single indicator, and could effectively identify high-risk AMI patients (Figure 5D). To evaluate the incremental predictive value of CARMN, we constructed baseline Model A incorporating cTnI, LVEF, and CK-MB without CARMN. Model A yielded an AUC of 0.721 (95% CI: 0.639-0.804), with a bootstrap-corrected C-index of 0.618 after 1000 resamples. Its bootstrap-corrected calibration curve suggested acceptable overall performance yet a tendency to overestimate risk in the high-risk range (Supplementary Figure S1). We further assessed the incremental prognostic value of CARMN using time-dependent IDI and NRI analyses at the 6-month endpoint. Relative to Model A, Model B produced an IDI of 0.068 (95%CI: 0.005-0.119, *P* = 0.028) and a continuous NRI of 0.196 (95%CI: 0.023-0.444, *P* = 0.024).

**Figure 5:**
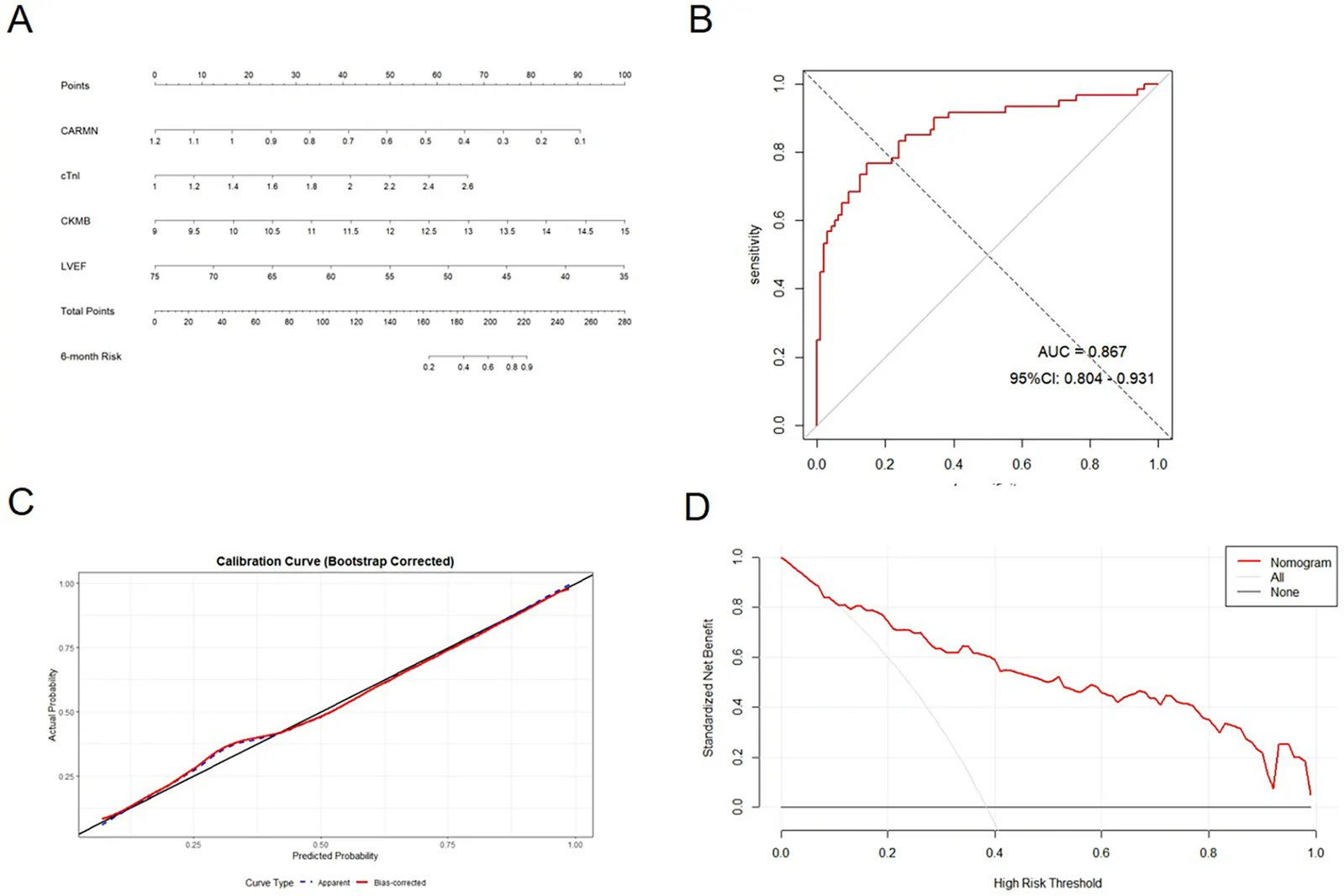
Construction and validation of a 6-month risk prediction nomogram model for MACE in patients with AMI. **(A)** Nomogram model. **(B)** ROC curve of the nomogram for predicting 6-month MACE. The AUC was 0.867 (95% CI: 0.804-0.931). **(C)** Nomogram calibration curve. **(D)** Decision curve analysis (DCA).

## Discussion

AMI is a severe coronary artery disease caused by the rupture of coronary artery plaques (27). Although PCI is the mainstay of current treatment, patients remain at a substantial risk of subsequent MACE, which is potentially life-threatening (28, 29). Therefore, there is an urgent need to identify easily detectable, highly specific biomarkers for accurate risk stratification of MACE in AMI patients. This study included 156 AMI patients. We measured serum CARMN levels and analyzed their associations with traditional myocardial injury markers and echocardiographic parameters (LVEF). Furthermore, we developed a prognostic nomogram integrating CARMN with these conventional indicators to facilitate individualized risk assessment for post-AMI MACE.

CARMN was initially discovered in human cardiac muscle cells and was named cardiac mesoderm enhancer-related non-coding RNA (30). The dysregulation of CARMN has been proven to be associated with various cardiovascular diseases (31, 32). For instance, Ni et al. discovered that CARMN is a key regulatory factor for the plasticity of vascular smooth muscle cells and the development of atherosclerosis (21). In mice with abdominal aortic aneurysms, the serum CARMN level was significantly lower than that in the healthy control group (33, 34). Our research found that CARMN is downregulated in AMI patients. This differential expression can be utilized to distinguish AMI patients from healthy controls. Nevertheless, this observation comes from a case-control comparison and has not been verified in suspected-AMI populations. This study investigated the expression level of CARMN in the prognosis of AMI patients. Kaplan–Meier analysis results showed that the incidence of MACE in patients with low CARMN expression was significantly higher. This indicates that CARMN may serve as a potential predictive indicator for evaluating the prognosis of AMI patients.

Previous studies have established significant associations between non-coding RNA (ncRNA) and both myocardial injury markers and echocardiographic parameters, providing an important theoretical basis for integrating ncRNAs with these clinical indicators in AMI (35, 36). For example, the expression of serum exosome lncRNA LINC00472 was significantly elevated in AMI patients, and it was significantly positively correlated with the levels of myocardial injury markers CK-MB and cTnI (18). The expression level of lncRNA APF was also significantly correlated with myocardial injury markers such as cTnI and CK-MB (37). Our results showed that CARMN was negatively correlated with myocardial injury markers such as cTnI, CK-MB, Myo, and positively correlated with LVEF. In addition, this study constructed a nomogram predict MACE risk in AMI patients within 6 months after PCI by combining CARMN with LVEF, cTnI, CK-MB. Based on internal validation of the self-service method, calibration curves and DCA analysis, this model demonstrates excellent calibration performance and significant clinical net benefits. It is worth noting that these results are still preliminary and require external validation before they can be confirmed. Additionally, the duration of ischemia from the onset of the disease to PCI in the patients included in this study varies individually, which may affect the accuracy of the biomarker detection results.

This study still has certain limitations. Firstly, this study is a single-center study with a short follow-up duration of 6 months. Therefore, the sample size and the number of endpoint events were limited. Secondly, the established nomogram was not directly compared with well-validated risk scoring systems (such as the GRACE score, TIMI score or simplified clinical models). The model was obtained solely through the use of six variables and internal validation using the bootstrap method. Thus, this prediction model has the risk of overfitting and can only be regarded as an exploratory and hypothesis-generating preliminary model. It is not yet supported for clinical decision-making applications. Further multi-center and large-sample studies are needed to complete external validation and conduct comparative analysis with existing risk scoring systems. In addition, this study did not deeply explore the specific molecular mechanisms by which CARMN regulates the occurrence, development and prognosis of AMI. In the future, we will conduct basic experiments to clarify the mechanism of CARMN and provide new molecular targets for the prevention and treatment of AMI.

## Conclusion

This study explored the potential value of CARMN for diagnosis and prognostic assessment in AMI. We constructed a nomogram integrating serum CARMN with conventional myocardial injury biomarkers (cTnI, CK-MB) and echocardiographic LVEF to predict MACE risk among AMI patients. This model shows promise for early risk stratification and may provide references for developing individualized management strategies for high-risk patients.

## Data Availability

The original contributions presented in the study are included in the article/Supplementary Material, further inquiries can be directed to the corresponding author.
