# Supplementary material for: Circulating CARMN for acute myocardial infarction prognosis and its incremental effect when combined with conventional biomarkers
Source: Front Cardiovasc Med. 2026 Oct 21;13:1881009. doi: 10.3389/fcvm.2026.1881009 (PMC13730833; doi:10.3389/fcvm.2026.1881009)
Supplement: Supplementary file 1 [file Table1.docx]

**Supplementary Table 1**. Information about PCI in patients with AMI

| Items | Patients with AMI (n = 156) |
| --- | --- |
| Reperfusion timing, median [IQR] |  |
| **Symptom‑to‑balloon time,** min | 223 [182-276] |
| **Door‑to‑balloon time,** min | 84 [66-102] |
| Medication**, n (%)** |  |
| β-receptor blockers | 117（75.00%） |
| Statins | 148（94.87%） |
| Antiplatelet drugs | 151（96.79%） |
| ACEI/ARB | 112（71.79%） |
| Calcium Channel blocker | 28（17.94%） |
| Infarct-associated arteries**, n (%)** |  |
| LMCA | 10 (6.41%) |
| LAD | 63 (40.38%) |
| LCX | 51 (32.69%) |
| RCA | 32 (20.52%) |

Abbreviations: PCI, percutaneous coronary intervention; AMI, acute myocardial infarction; LMCA, left main coronary artery; LAD, left anterior descending; LCX, left circumflex coronary artery; RCA, right coronary artery.
